# Clinical evaluation of a machine learning-based dysphagia risk prediction tool

**DOI:** 10.1007/s00405-024-08678-x

**Published:** 2024-05-14

**Authors:** Markus Gugatschka, Nina Maria Egger, K. Haspl, David Hortobagyi, Stefanie Jauk, Marlies Feiner, Diether Kramer

**Affiliations:** 1 Department of Phoniatrics, ENT University Hospital Graz, Medical University Graz, Graz, Austria; 2 Steiermärkische Krankenanstaltengesellschaft m.b.H. (KAGes), Graz, Austria; 3 PH Predicting Health GmbH, Graz, Austria

**Keywords:** Dysphagia screening, Machine learning, Real time evaluation

## Abstract

**Purpose:**

The rise of digitization promotes the development of screening and decision support tools. We sought to validate the results from a machine learning based dysphagia risk prediction tool with clinical evaluation.

**Methods:**

149 inpatients in the ENT department were evaluated in real time by the risk prediction tool, as well as clinically over a 3-week period. Patients were classified by both as patients at risk/no risk.

**Results:**

The AUROC, reflecting the discrimination capability of the algorithm, was 0.97. The accuracy achieved 92.6% given an excellent specificity as well as sensitivity of 98% and 82.4% resp. Higher age, as well as male sex and the diagnosis of oropharyngeal malignancies were found more often in patients at risk of dysphagia.

**Conclusion:**

The proposed dysphagia risk prediction tool proved to have an outstanding performance in discriminating risk from no risk patients in a prospective clinical setting. It is likely to be particularly useful in settings where there is a lower incidence of patients with dysphagia and less awareness among staff.

## Introduction

Dysphagia is a very common but often underdiagnosed condition. Available data vary considerably, but epidemiologic surveys show that dysphagia was present in 2.4% of all hospital discharges and was more frequent in older patients and in men. Higher prevalence of dysphagia was found in acute geriatric units (10.3%), neurology (7.6%) and internal medicine (7.5%) wards [1]. The large variability in prevalence data results from the different frequencies in the age groups, different assessment tools used for diagnosis and clinical expression, and the patient environment (in- or out-patients), among others. Dysphagia is silent in many cases and is often detected for the first time in elderly patients after hospital admission for concomitant conditions, without it being the main cause of hospitalization. It is associated with higher risk of aspiration pneumonia and a consecutively increased risk of prolonged hospital stays and mortality risk [2].

The available diagnostic tools are often time and personnel intensive, which probably leads to a high rate of cases not diagnosed in time. At the same time, many countries are experiencing unprecedented shortages in nursing professions. Modern IT-technology can provide important assistance here.

The increasing digitization of patient data and medical records has enabled the widespread application of machine learning (ML) and deep learning technologies in recent years. The advantages of being able to capture and process large amounts of data have led to better diagnostics and also a path towards personalized medicine. In recent years, a number of ML algorithms have been developed that have found applications in various fields such as early detection of delirium [3], radiologic diagnostics [4], prediction of survival times [5] and many more.

We recently published first results from a prospective trial where we evaluated the performance of a ML-based dysphagia prediction tool in two different cohorts including 1270 patients. The discriminative performance was excellent with an area under the receiver operating-characteristic curve (AUROC) of 0.841, a sensitivity of 74.2%, and a specificity of 84.1% [6]. A limitation of this study was that results were based on routinely assessed data only. In the present study, data from the risk prediction tool were supplemented with additional and specific clinical evaluations and diagnoses from medical experts.

## Material and methods

### Dysphagia risk prediction tool

The dysphagia risk prediction tool is a machine learning (ML)-based software that assesses the individual risk of patients in hospitals, nursing homes and private practices in order to better target available resources (especially for preventive treatment). It was developed by a team of technical and clinical experts within and outside of the regional health care provider in Styria (Austria), the Steiermärkische Krankenanstaltengesellschaft m. b. H. (KAGes). For individual patients, the software calculates the individual risk of the occurrence of a disease or syndrome, a complication, an adverse condition or a clinically relevant event (e.g. intensive care stay after an operation) for a certain period of time. For this study, a previously designed model for predicting dysphagia was integrated in the Personalized Risk Tool and implemented the hospital information system of the ENT University Hospital Graz openMEDOCS (i.s.h.med Cerner corp., MI, USA) [6].

The integrated model was trained on electronic health record (EHR) data of 33,784 in-patients, who had been admitted to KAGes hospitals between 2011 and 2019. More than 800 prediction features were built, based upon longitudinal patient histories using routinely documented diagnoses, procedures, laboratory values, nursing data, medication, demographic and administrative data. As mentioned above, the risk prediction tool, trained with the random forest method, achieved an AUROC of 0.94 on unseen test data [7], and an AUROC of 0.841 under prospective evaluation in an internal medicine department [6].

### Clinical evaluation

In a real time evaluation setting, as the current one is, the prediction of dysphagia by the software was performed automatically for every patient admitted; an HL7 message was sent from the hospital information system (HIS) to a local hospital server, patient data needed for prediction were retrieved using http-requests. The prediction tool running on the server predicted the risk of dysphagia for each patient at (1) admission time, (2) the evening of admission and (3) the second evening. Overnight recalculations were performed to include the most recent laboratory results and nursing assessment data. Patients were classified in *high risk*/*very high risk* and *no risk* groups. All risk predictions and features values were stored in a data warehouse.

The results were furthermore checked daily by the study team (four speech language specialists). Cases of the *high* and *very high* risk group, as classified by the risk prediction tool, underwent further clinical investigation. For this purpose, a comprehensive review based on the same medical records that were taken into consideration by the risk prediction tool was carried out. Subsequently these were either classified as patients at risk/no risk.

Data review included analyzing results of clinical swallowing examinations, fiberendoscopic swallowing examinations (FEES), video swallowing X-rays, as well as current medical summaries, interprofessional documentation (by nurses, SLP, dietetics, physiotherapy etc.). Where necessary, further treatment was initiated.

Additionally, the team checked the entire occupancy list of the ward, i.e. patients where the algorithm did not detect a risk. This included the review of diagnoses and comments about swallowing ability, which were reviewed in the interprofessional documentation section.

### Data analysis

Univariate and bivariate analyses were carried out to describe the cohort. In addition to age and gender, the primary diagnoses of admission were considered. Primary diagnoses were grouped and summarized: (1) *oropharyngeal malignancies*, (2) *inflammatory diseases of the head and neck* (erysipelas, herpes infection etc.), (3) *acute/chronic affections of nose/sinuses*, (4) *acute/chronic affections of ear incl. middle and inner ear* (vertigo, hearing loss, chronic otitis etc.), (5) *affections of the salivary glands* (benign and malign affections), (6) *acute/chronic affections of oral cavity* (tonsillitis, peritonsillar abscess, cervical abscess etc.) and (7) *other* (fractures, burn injuries etc.).

### Performance of the risk prediction tool

The performance of the risk prediction tool was described using discrimination and calibration measures. Receiver operating characteristic (ROC) curves with DeLong confidence intervals [8] and AUROC values were used as measures of discrimination. Confidence intervals (95%) were calculated with 2000 stratified bootstrap replicates using the R pROC package [9]. According to Hosmer, an AUROC value above 0.7 is interpreted as acceptable, a value above 0.8 as excellent and above 0.9 as outstanding discrimination [10]. Furthermore, we have calculated sensitivity, specificity, positive predictive value (precision), negative predictive value and accuracy. To measure calibration, a calibration plot with a 95% confidence interval was constructed. This plot illustrates the relationship between the observed and predicted frequency of dysphagia patients.

## Results

Demographic data and primary diagnoses according to clinical evaluation are displayed in Tables 1 and 2. During the observation period 149 inpatients of the ENT department were screened (93 men, 56 women). The ML algorithm identified 44 patients (29.5%) at high or very high risk resp. 105 (70.5%) patients at no risk. On the other side, 51 (34.2%) were identified as patients at risk by the medical experts (see confusion matrix Table 3).

**Table 1:** Demographic distribution classified by risk/no risk

	n	Clinical evaluation	
		No risk	Risk
Age, years		51.2 (32.4–70.1)	57.3 (39.9–74.7)
Sex (n)	Male	56 (60.2%)	37 (39.8%)
	Female	42 (75,0%)	14 (25,0%)

**Table 2:** Primary outcome diagnoses classified clinically by risk/no risk

	Clinical evaluation	
	No risk	Risk
1. Oropharyngeal malignancies	14 (14.3%)	29 (56.9%)
2. Inflammatory diseases of the head and neck	4 (4.1%)	1 (2,0%)
3. Acute/chronic affections of nose/sinuses	33 (33.7%)	0 (0,0%)
4. Acute/chronic affections of ear incl. middle and inner ear	18 (18.4%)	0 (0,0%)
5. Affections of the salivary glands	4 (4.1%)	1 (2,0%)
6. Acute/chronic affections of oral cavity	8 (8.2%)	9 (17.6%)
7. Other	17 (17.3%)	11 (21.6%)

Percentages are displayed column wise

**Table 3:** Confusion matrix

	Prediction				Total	
	No risk		Patients at risk (high/very high risk)			
	*n*	%	*n*	%	*n*	%
Clinical evaluation						
No risk	96	**98.0**	2	2.0	98	100.0
Risk	9	17.6	42	**82.4**	51	100.0
Total	105	70.5	44	29.5	149	100.0

Percentages are displayed row wise. 98 = Specificity and 82.4 = Sensitivity

**Fig. 1:**
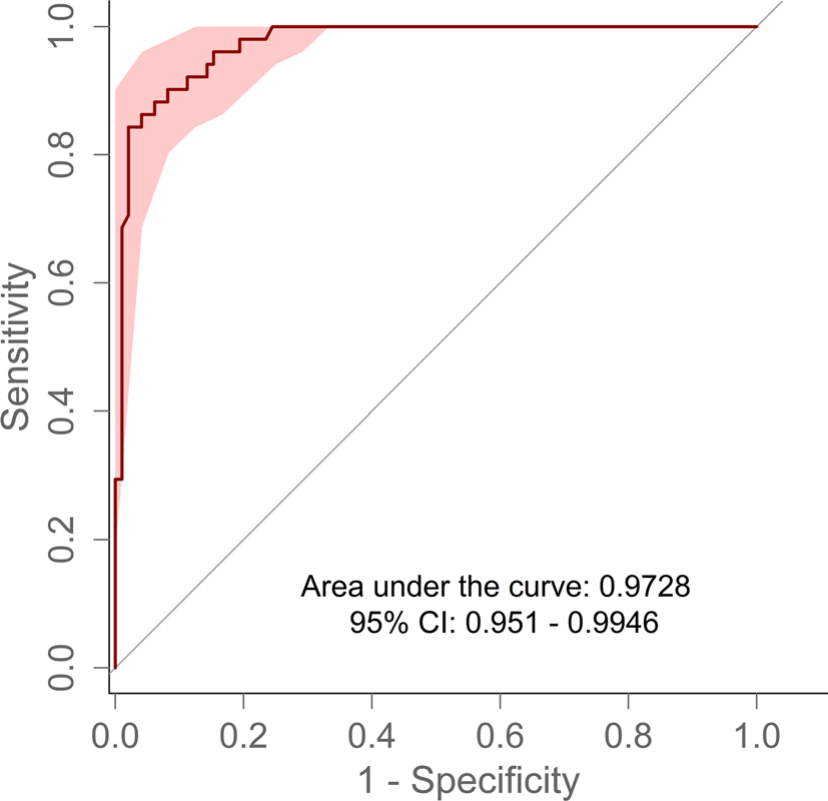
ROC for validation data

Figure 1 shows the ROC for the validation data. The AUROC for this cohort was 0.9728 [0.9510–0.9946]. In 96 cases (64.3%) neither the clinicians nor the algorithm identified any risk of dysphagia. This led to a specificity of 98.0% [90.8–99.9%]. On the other hand, the sensitivity in this cohort achieved 82.4% [31.4–92.2%], with 42 correctly identified cases of dysphagia out of a total of 51. The accuracy achieved a value of 92.6% [87.9–93.9%], the positive predictive value (precision) was 95.5% [88.9–95.9%], and the negative predictive value 91.6% [90.8–91.6%].

**Fig. 2:**
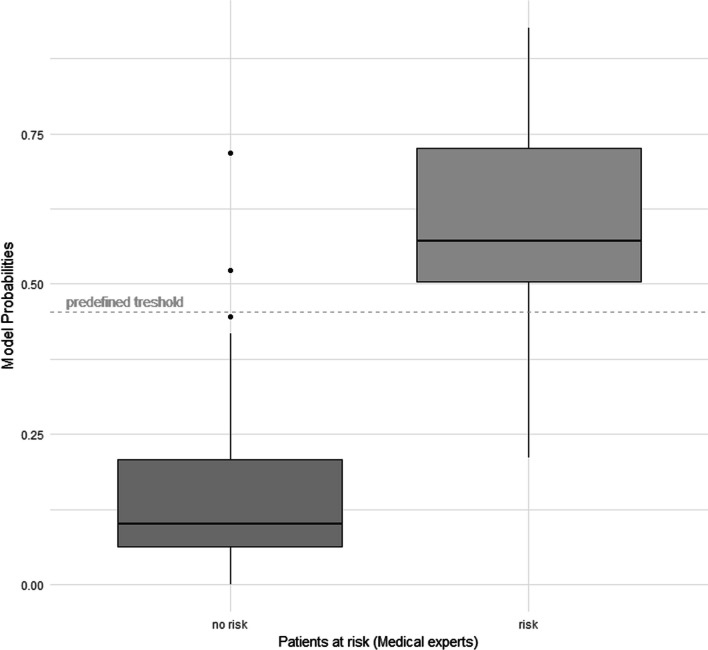
Distribution of the model's risk probabilities for the two categories classified by medical experts. The predefined threshold shows the stratification boundary of the model

To analyze the discrimination in more detail, the predicted probabilities can be seen in Fig. 2. The distribution of probabilities shows that the two classes can be distinguished clearly (risk/no risk). Based on the distribution of the calculated probabilities, the prediction tool will always show good discrimination if a cut-off value is selected in the range between the two boxes. In this specific range, any cut-off value will render sensitivity and specificity values above 75%.

**Fig. 3:**
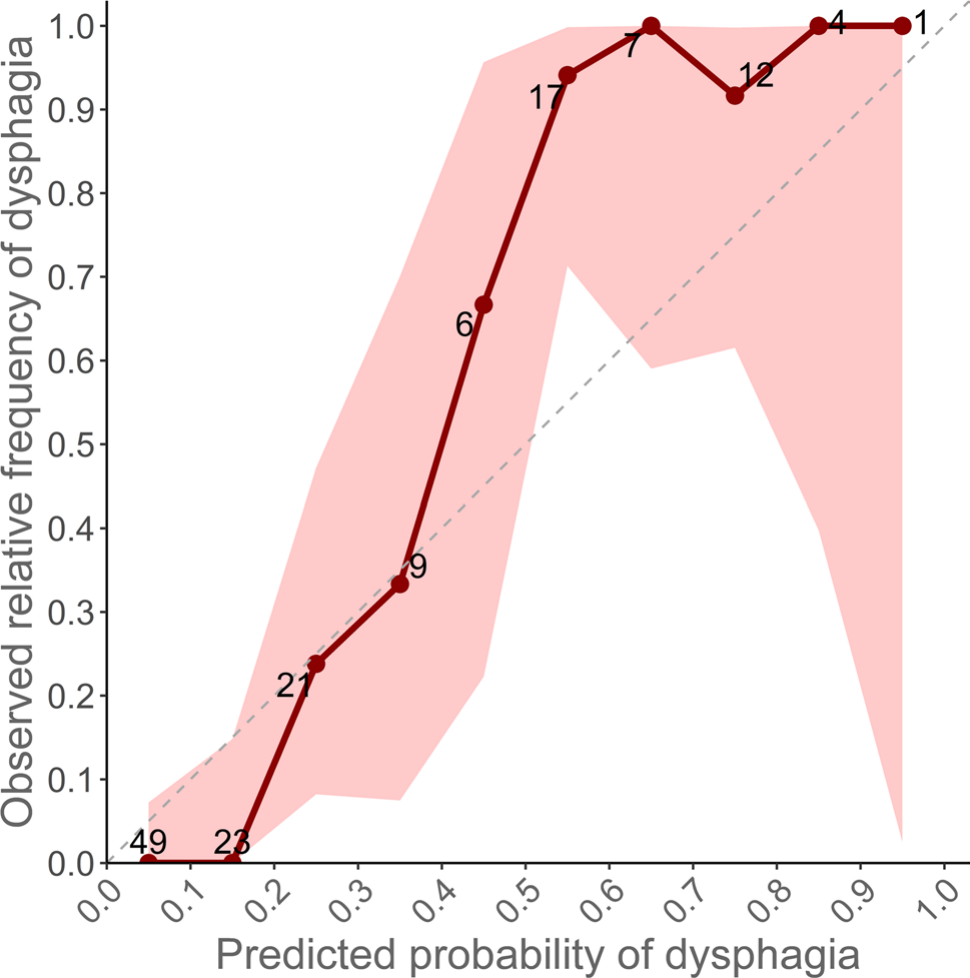
Calibration plot

The calibration plot is presented in Fig. 3. The plot shows a slight overestimation of the dysphagia risk when compared to the observed frequency of the outcome. Due to the small number of observations, the confidence interval broadens the higher the estimated probability.

The group of patients as classified of having a risk by the medical experts was significantly older, 51.2 years vs. 57.3 years (*p* = 0.05). More patients in the risk group had an oro-pharyngeal malignancy (56.9% vs. 14.3%). About a third of the study population was hospitalized for elective surgery due to acute or chronic affections of nose/sinuses or ear including middle and inner ear. As expected, for this group neither the algorithm nor the medical experts identified patients at risk.

## Discussion

As in many countries, the shortage of healthcare professionals is increasing the need for reliable and validated decision support tools. Automated screening tools can be a good support and have the advantage of not requiring additional effort to obtain a personalized risk assessment. The underlying algorithms are based on existing data, which in the medical context come from electronic health records (EHRs). These comprise data from laboratories, medication, nursing and medical documentation etc. Naturally these data do not have the same quality as data collected for e.g., clinical trials, but this is compensated by the large amount of data processed [11]. Based on this approach we were able to demonstrate in previous works that very good risk assessments can be achieved and that the users did not have any additional workload for the screening [3, 7].

As shown in a previous paper the same ML-based risk prediction software achieved an excellent performance in predicting dysphagia in a cohort of 1270 inpatients solely based on routinely documented outcome classification in a more general internal and geriatric ward [6]. However, we assumed that by using this approach we might have missed a considerable number of patients at risk. To close this gap, we matched ML generated risk profiles with clinical investigations and assessment from medical experts in an ENT department. By this, we sought to determine the reliability and trustworthiness of the software in clinical routine, a step that is inevitable before expanding to larger cohorts. Incorrect decisions of the algorithm can lead to serious consequences for the patients. At the same time, the operability must be intuitive for different professional groups treating the patients.

By comparing the pre-trained algorithm with the evaluation by the medical experts (gold standard) we achieved an excellent area under the curve of 0.9728 (95% CI 0.9510–0.9946), which outperforms the results of our precedent study (AUROC of 0.841, CI 0.7781–0.9046). This is based on the more accurate classification of the outcome variable compared to labelling based on routine data (EHR) as pursued previously [6]. In particular, all patients at risk were classified by the medical experts which made it possible to delineate the grey area of false positives much better.

Our data showed that there were relatively more men (39.8%) at risk than women (25%). This is in accordance with literature. A systematic review by Rajati et al. reported a prevalence for oropharyngeal dysphagia of 54.7% in men and 46.5% in women [12]. In addition, it is known that men are more susceptible to head and neck cancers [13]. This group, of course, contributes significantly to the occupancy of an ORL ward (see Table 2). On the other hand, the risk of dysphagia was low in patients hospitalized for elective nose, sinus, and ear procedures.

In 11 of the 149 cases, data of the algorithm and the clinical assessment did not match. In two cases the algorithm calculated a risk of dysphagia, which was not confirmed by the medical experts. This might be explained by the fact that the algorithm also included diagnoses related to dysphagia which were made a long time ago but were revised in the meantime. According to the medical records one of these patients had a diagnosis of dysphagia made in 2010. In the meantime the patient had undergone dysphagia therapy by a speech therapist and was free of OD symptoms since this.

In nine cases where an increased risk was diagnosed by clinical assessment, the results of the algorithm did not match. Like above, these discrepancies maybe due to different factors. Dysphagia is a highly complex condition where the entire individual medical history needs to be considered.

The risk prediction tool could only rely on data that were available in the hospital information system. Outpatient visits to (specialist) physicians in private practice, discharge documents from other hospitals etc. were not included in the calculation, which might have led to inconclusive results. Also, due to the complex structure of the hospital information system, it may happen that certain relevant information (e.g. free text fields of certain electronical documents, etc.) was not included in the calculation.

The results of our study are influenced by the specific cohort at an ENT department. Diagnoses such as oropharyngeal malignancies occur more frequently at an ENT clinic, while simultaneously the staff are more aware of swallowing disorders. The software is likely to be particularly useful primarily in institutions where there is a lower incidence of patients with dysphagia and less awareness. The characteristics of our cohort with a high homogeneity compared to e.g. patients at an internal medicine ward, poses a high challenge to the algorithm. Regardless of this, we were able to improve the algorithm.

## Conclusion

To the best of our knowledge this is the first time a ML based dysphagia risk prediction tool was validated in real time in a clinical setting. The tool is not intended to replace the diagnostic process, but rather to draw attention to an increased risk of dysphagia. In an intensive 3-week validation phase, 149 patients were screened automatically in real time and results were compared with the assessments of medical professionals. Based on a considerable amount of data, the algorithm proved to be an excellent tool in discriminating patients at risk vs. no risk. Augmenting the algorithm with clinical data from each single patient in the cohort, the AUROC outperformed the results of a previous study. We believe our software can be a useful tool for screening large numbers of patients in real time. The next steps will be to create decision processes for how the information is handled.

## Data Availability

Not applicable.
